# Diagnostic and prognostic value of PCT and RDW in premature infants with septicemia

**DOI:** 10.1097/MD.0000000000035725

**Published:** 2024-02-16

**Authors:** Huafen Xu, Dong Ca, Lixia Zhou

**Affiliations:** a Department of Neonatology, Hainan Provincial People’s Hospital, Haikou, Hainan, China.

**Keywords:** PCT, premature infant, RDW, septicemia

## Abstract

It aims to study the diagnostic effect of procalcitonin (PCT) and red blood cell distribution width (RDW) in premature septicemia (PS), and to analyze the prognostic evaluation value of PCT and RDW in PS. Ninety eight septicemia premature infants (SPI) who visited the neonatal intensive care unit of our hospital from June 2019 to July 2021 were selected and met the criteria. Based on the patient’s condition and the neonatal shock score, they were separated into a severe group (SG) and a mild group (MG). There were 43 children and 55 children in the 2 groups, respectively. According to the survival status of SPI after 3 days of treatment, they were divided into a death group and a SG. It detected and analyzed the peripheral venous blood of SPI before treatment (BT) and after treatment (AT), and observed the changes of PCT and RDW. The comparison of general data between severe and mild SPI and their mothers did not have statistical significance (*P* > .05). The PCT of the SG was higher than that of the MG BT, on the 1st day and the 3rd day AT; The PCT BT and AT in both groups ranged from high to low on the 1st day and the 3rd day AT and BT (*P* < .05). The RDW in the SG were higher than those in the MG, and the RDW BT and AT in both groups were the highest on the 1st day AT; The RDW BT in the MG was higher than on the 3rd day AT, while the RDW BT in the SG was lower than on the 3rd day AT (*P* < .05). The optimal cutoff values for PCT on the 1st and 3rd day AT were 40.594ng/ml and 64.854ng/ml, respectively, with sensitivity of 100.0% and 100.0%, and specificity of 73.2% and 87.1% (*P* < .05). The optimal cutoff values for RDW on the 1st and 3rd day AT were 16.649% and 18.449%, respectively, with sensitivity of 100.0% and 100.0%, and specificity of 68.5% and 91.8% (*P* < .05). Monitoring the changes in PCT and RDW can promote the early diagnosis of PS and their prognosis evaluation.

## 1. Introduction

Premature infants (PI) refer to newborns under 37 weeks of gestational age (GA). They are a special type of newborn because their various organs and tissues are immature and have poor living abilities. So they are likely to experience various short-term and long-term complications after birth, which is also an important factor leading to perinatal death and long-term disability. The incidence of PI in China is approximately 5% to 10%. Especially with the relaxation of the second child policy, high-risk pregnant women have significantly increased, and the incidence of premature birth is on the rise. With the rapid development of perinatal care and neonatal critical care, the survival rate of PI is increasing year by year. At present, the success rate of rescue for extremely low weight infants both domestically and internationally exceeds 90%, and the survival rate over 26 weeks is even more than 70%. As the growth of medical technology, the success rate and survival rate of rescue for PI, especially extremely PI, have greatly improved.^[2,3]^ However, due to poor adaptability to environmental changes and immature development of multiple organs, PI are prone to various complications. Therefore, how to implement more reasonable and effective comprehensive treatment, thereby improving the prognosis of PI and improving their quality of life, has become the focus of modern neonatologists attention. Procalcitonin (PCT) is a hormone free precursor of calcitonin, which is synthesized by thyroid C cells. In normal human body, its PCT concentration is generally < 0.1 ng/mL. When systemic inflammatory reactions occur, macrophages and monocytes in the liver, as well as lymphocytes in lung and intestinal tissues, also produce a large amount of PCT, leading to an increase in serum PCT concentration. Under normal circumstances, PCT greater than or equal to 0.5 ng/mL is considered normal. If severe bacterial infections occur, PCT can reach 1000 ng/mL.^[5,6]^ At present, the clinical application of PCT in premature septicemia (PS) and severe bacterial infections has been widely confirmed, but there is currently a lack of relevant research on newborns. Distribution width (RDW) refers to the amount of change in red blood cell volume, and is an important indicator reflecting the heterogeneity of it in blood routine. Due to its easy accessibility, it has been extensively applied in the diagnosis and differentiation of various anemia diseases.^[7,8]^ RDW poses a serious threat to human health, including coronary heart disease, acute myocardial infarction, and acute pulmonary embolism.^[9]^ In recent years, studies have found that high RDW is also associated with death in patients. Due to the relationship between PCT and RDW and the severity and outcome of infection, there is currently no research on the early diagnosis and outcome of PS using these 2 indicators. Therefore, this study will use PCT and RDW to monitor the changes in PS, thereby promoting its diagnosis and prognosis.

## 2. Objects and methods

### 2.1. Research objects

This study was approved by the Ethics Committee of Hainan Provincial People’s Hospital. It randomly selects septicemia premature infants (SPI) who visited the neonatal intensive care unit of our hospital from June 2019 to July 2021, and the inclusion criteria are as follows: Diagnosed as PI; PI have a GA of < 37 weeks and a birth weight (BW) of < 2.5 kg; PI should be < 72 hours old at admission; No septicemia within 72 hours of age; The guardians of PI agree to join in the research and sign an informed consent notice.^[10,11]^ The exclusion criteria are as follows: Suffering from genetic metabolic diseases; Contamination of amniotic fluid and meconium; Patients with incomplete liver and kidney function; Those who are not of GA; Twins or multiple births; Suffering from diseases that cause changes in red blood cells. After screening, 98 eligible patients are obtained and divided into severe group (SG) and mild group (MG) based on the patient’s condition and the neonatal shock score. The shock score of the SG is above 6 points, while the shock score of the MG is below 6 points. There are 43 children and 55 children in the 2 groups, respectively. According to the survival status of SPI after 3 days of treatment, they are classified into death group (DG) and SG, with 11 and 87 cases, respectively.

### 2.2. Research methods

Statistics were conducted on the age and pregnancy complications of 98 mothers of SPI at delivery, as well as the gender, GA, BW, and birth mode of SPI; and it needs to record the PCT and RDW at the first examination 72 hours after birth for SPI. It also needs to compare the general data of SG, MG, dead group and SG, detect the peripheral venous blood of SPI before treatment (BT) and after treatment (AT), use the automatic blood analyzer to observe and analyze the changes of PCT and RDW of 4 groups of SPI.^[12,13]^

### 2.3. Outcome measures

It includes the gender, GA, BW, birth mode, PCT and RDW at the first examination 72 hours after birth of SPI, age at delivery and complications during pregnancy of their mothers and PCT, RDW, and other related indicators BT and AT for PS.

### 2.4. Statistical methods

It uses IBM SPSS Statistics 26.0 software to perform statistical and analytical processing on the data. If the measurement data in the study conforms to the normal distribution, it is presented in the form of mean ± standard deviation $\left(\overset{-}{x}\;\pm s\right)$, and the t test is used for inter group comparison.^[14]^ If not, it uses $M\left(P_{25}∼P_{75}\right)$ for representation and Mann Whitney U test for Mann–Whitney *U* testthe 2 groups; Friedman test is used for skewed distribution data related to multiple groups; Pairwise comparison uses Wilcoxon signed rank sum test.^[15]^ The counting data is expressed in frequency or percentage form, and $\chi^{2}$ test is utilized for inter group comparison. Receiver operating characteristic (ROC) curves are used to predict the expression of PCT and RDW at different periods. The significance level is taken as 0.05, and if *P* < .05, it denotes statistical s*i* < ificance.

## 3. Results

### 3.1. Comparison of general information between severe and mild SPI

It compares the general information of SPI between the SG and MG, including GA, BW, gender, birth method, and Apgar scores at 1 and 5 minutes. Among them, *P* > .05, indicating no significant difference. The comparison of general information is shown in Table 1.

**Table 1:** General information of the severe and mild SPI.

Group		SG	MG	*t* or $\chi^{2}$	*P* value
Number of cases		43	55	/	/
GA ($\overset{-}{x}\;\pm s$, week)		32.4 ± 2.0	32.2 ± 1.7	1.076	.284
BW ($\overset{-}{x}\;\pm s$, g)		1701 ± 299	1599 ± 301	1.625	.109
Male (case, %)		25 (58)	33 (60)	0.336	.564
Cesarean delivery (case, %)		26 (60)	30 (55)	0.324	.570
Apgar score ($\overset{-}{x}\;\pm s$, score)	1 min	8.2 ± 0.9	8.5 ± 1.2	−0.388	.699
	5 min	9.4 ± 0.9	9.1 ± 0.6	0.306	.760

GA = gestational age, MG = mild group, SG = severe group, SPI = 21epticaemia premature infants.

**Figure 1.**
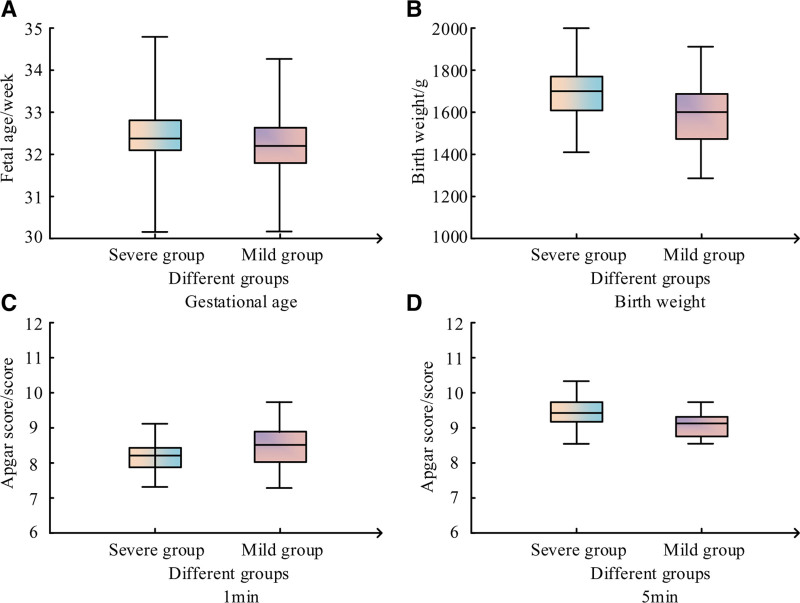
General situation of the severe and mild SPI. SPI = septicemia premature infants.

The average GA of the severe and mild SPI was (32.4 ± 2.0) weeks and (32.2 ± 1.7) weeks, respectively; The average BW was (1701 ± 299) g and (1599 ± 301) g, respectively. The Apgar scores of the 2 groups at 1 minute were (8.2 ± 0.9) and (8.5 ± 1.2), respectively; The Apgar scores at 5 minutes were (9.4 ± 0.9) and (9.1 ± 0.6), respectively, as shown in Figure 1.

### 3.2. Comparison of general information between mothers of severe and mild SPI

Comparison of the general information of mothers of severe and mild sepsis PI includes PCT and RDW at the first examination after 72 hours of age, maternal age, and pregnancy complications. The PCT at the first examination after 72 hours of age in the SG and MG of SPI were (0.5 ± 0.3) ng/mL and (0.6 ± 0.3) ng/mL, respectively; The RDW were (15.8 ± 2.0)% and (16.2 ± 2.3)%, respectively. Among them, PCT and RDW at the first examination after 72 hours of age, maternal age, and pregnancy complications were all *P* > .05, without significant differences. The general comparison of data is shown in Table 2.

**Table 2:** General information of mothers of severe and mild SPI.

Group		SG	MG	*t* or $\chi^{2}$	*P* value
Number of cases		43	55	/	/
First examination after 72 h of age ($\overset{-}{x}\;\pm s$)	PCT (ng/mL)	0.5 ± 0.3	0.6 ± 0.3	−0.649	.326
	RDW (%)	15.8 ± 2.0	16.2 ± 2.3	−0.746	.459
Maternal age ($\overset{-}{x}\;\pm s$, age)		29.9 ± 4.9	30.6 ± 4.8	−0.422	.676
Complications during maternal pregnancy (case, %)	Gestational diabetes	6 (14)	6 (11)	0.259	.609
	Hypertensive disorder complicating pregnancy	11 (26)	10 (18)	0.760	.385

MG = mild group, PCT = procalcitonin, RDW = distribution width, SG = severe group, SPI = 22epticaemia premature infants.

### 3.3. Comparison of PCT and RDW levels in severe and mild SPI at different stages

Compared with the MG, the PCT of the SG was higher BT, on the 1st day and the 3rd day AT; The PCT BT and AT in both groups ranged from high to low on the 1st day and the 3rd day AT, and BT (*P* < .05). The PCT in the SG BT, on the 1st day and the 3rd day AT were 3.8 ng/mL, 43.2 ng/mL, and 26.2 ng/mL, respectively; The PCT in the MG BT, on the 1st day and the 3rd day AT were 1.5 ng/mL, 17.2 ng/mL, and 24.7 ng/mL, respectively. On the 1st day and 3rd day AT, the RDW in the SG were higher than those in the MG. Among the 2 groups, the RDW BT and AT were the highest on the 1st day AT; The RDW BT in the MG was higher than on the 3rd day AT, while the RDW BT in the SG was lower than on the 3rd day AT (*P* < .05). The RDW in the SG were 16.6% and 16.2% on the 1st and 3rd days AT, respectively. The RDW in the MG were 16.4% and 15.8% on the 1st and 3rd days AT, respectively. The specific results are shown in Table 3.

**Table 3:** Levels of PCT and RDW in severe and mild SPI at different stages.

Group		SG	MG	*Z*	*P* value
Number of cases		43	55	/	/
PCT (ng/mL)	BT	3.8 (0.5–37.4)	1.5 (0.3–4.9)	−2.018	.045
	On the 1^st^ d AT	43.2 (18.6–82.9)*	17.2 (4.2–34.5)*	−3.349	0039
	On the 3th d AT	26.2 (3.9–67.4)†	4.7 (0.25–33.0)†	−2.931	.004
	$\chi^{2}$	14.334	34.546	/	/
	*P* value	.001	<.001	/	/
RDW (%)	BT	16.0 (15.5–17.3)	15.9 (15.2–17.5)	−0.409	.683
	On the 1^st^ d AT	16.6 (16.3–18.7)*	16.4 (15.6–17.2)*	−1.993	0047
	On the 3th d AT	16.2 (15.6–19.5)†	15.8 (15.2–16.6)†	−2.166	.029
	$\chi^{2}$	9.838	12.655	/	/
	*P* value	.006	.003	/	/

Compared with BT in the same group.

AT = after treatment, BT = before treatment, MG = mild group, PCT = procalcitonin, RDW = distribution width, SG = severe group, SPI = epticaemia premature infants.

* represents *P* < .05; Compared with the 1^st^ day AT in the same group.

† Indicates *P* < .05.

### 3.4. Comparison of general information between the SG and the DG of SPI

Comparison of the general information of SPI in the survival and DG includes GA, BW, gender, birth method, and Apgar scores at 1 and 5 minutes. Among them, *P* > .05, indicating no significant differences. The specific comparison of general information is shown in Table 4.

**Table 4:** General information of SPI in the SG and DG.

Group		DG	SG	*t* or $\chi^{2}$	*P* value
Number of cases		11	87	/	/
GA ($\overset{-}{x}\;\pm s$, wk)		31.6 ± 1.4	32.5 ± 1.9	1.176	.280
BW ($\overset{-}{x}\;\pm s$, g)		1629 ± 312	1663 ± 389	0.075	.787
Male (case, %)		9 (82)	49 (56)	1.789	.305
Cesarean delivery (case, %)		6 (60)	50 (57)	1.760	.745
Apgar score ($\overset{-}{x}\;\pm s$, score)	1 min	8.6 ± 1.2	8.0 ± 0.9	2.912	.090
	5 min	9.4 ± 0.9	9.4 ± 0.9	0.005	.936

DG = death group, GA = gestational age, SG = severe group, SPI = epticaemia premature infants.

**Figure 2.**
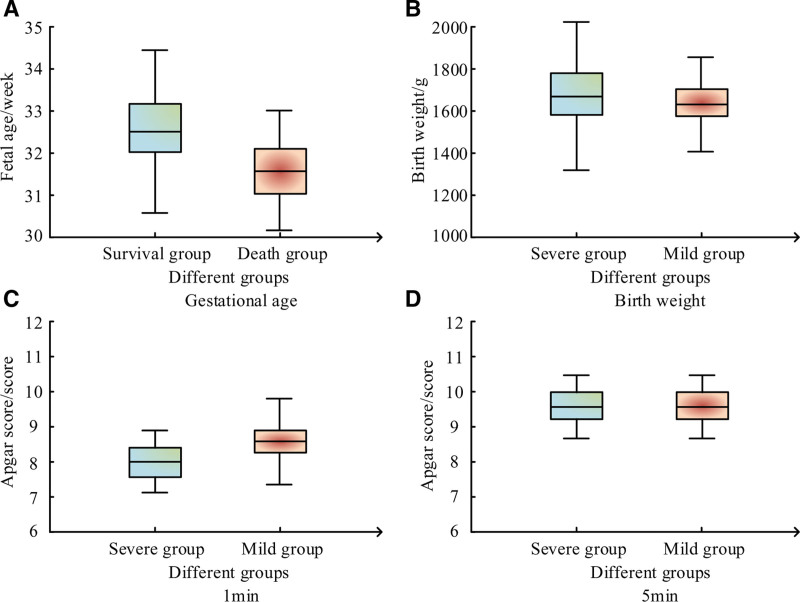
General situation of SPI in the SG and DG. DG = death group, SG = severe group, SPI = septicemia premature infants.

The average GA of SPI in the SG and the DG were (32.5 ± 1.9) weeks and (31.6 ± 1.4) weeks, respectively; The average BW were (1663 ± 389) g and (1629 ± 312) g, respectively. The Apgar scores of the 2 groups at 1 minute were (8.6 ± 1.2) and (8.0 ± 0.9), respectively; The Apgar scores at 5 minutes were (9.4 ± 0.9) and (9.4 ± 0.9), respectively, as shown in Figure 2.

### 3.5. Comparison of general information of mothers of SPI between SG and DG

Comparison of the general information of mothers of SPI in the SG and DG includes PCT and RDW at the 1st examination after 72 hours of age, maternal age, and pregnancy complications. The PCT of SPI in the SG and the DG were (0.6 ± 0.3) ng/mL and (0.6 ± 0.5) ng/mL at the 1st examination after 72 hours of maternal age, respectively; The RDW were (15.9 ± 2.2)% and (15.5 ± 1.8)%, respectively. Among them, PCT and RDW at the first examination after 72 hours of age, maternal age, and pregnancy complications were all *P* > .05, without significant differences. The specific comparison of general data is presented in Table 5.

**Table 5:** General information of mothers of SPI in the SG and DG.

Group		DG	SG	*t* or $\chi^{2}$	*P* value
Number of cases		11	87	/	/
First examination after 72 h of age ($\overset{-}{x}\;\pm s$)	PCT (ng/mL)	0.6 ± 0.5	0.6 ± 0.3	0.024	.880
	RDW (%)	15.5 ± 1.8	15.9 ± 2.2	0.650	.424
Maternal age ($\overset{-}{x}\;\pm s$, age)		30.9 ± 4.9	30.3 ± 4.7	0.112	.740
Complications during maternal pregnancy (case, %)	Gestational diabetes	4 (36)	12 (14)	2.129	.160
	Hypertensive disorder complicating pregnancy	4 (36)	17 (20)	0.734	.410

DG = death group, PCT = procalcitonin, RDW = distribution width, SG = severe group, SPI = epticaemia premature infants.

### 3.6. Comparison of PCT and RDW in SPI during different treatment periods between the SG and DG

Compared with the SG, the PCT and RDW in the DG were higher on the 1st and 3rd days AT than in the SG (*P* < .05); However, there was no statistically significant difference between the 2 groups BT (*P* > .05). The PCT levels BT and AT in both groups ranged from high to low on the 1st day and the 3rd day AT, and BT (*P* < .05). The PCT in the DG were 161.9 ng/mL, 122.7 ng/mL, and 12.2 ng/mL on the 1st day, 3rd day, and BT, respectively; The PCT in the SG were 20.0 ng/mL, 6.3 ng/mL, and 1.7 ng/mL on the 1st day, 3rd day, and BT, respectively. The RDW BT and AT in the DG ranged from high to low on the 3rd day and the 1st day AT and BT; The RDW in the SG BT and AT ranged from high to low on the 1st day AT, BT, and on the 3rd day AT (*P* < .05). The RDW in the DG were 21.9%, 18.5%, and 15.9% on the 3rd day, 1st day, and BT, respectively; The RDW in the SG on the 1st day, BT, and on the 3rd day AT were 16.2%, 15.8%, and 15.7%, respectively. The specific outcomes are shown in Table 6.

**Table 6:** PCT and RDW in SPI at different stages in the SG and DG.

Group		DG	SG	*Z*	*P* value
Number of cases		11	87	/	/
PCT (ng/mL)	BT	12.2 (0.4–82.4)	1.7 (0.4–14.4)	−1.309	.192
	On the 1^st^ d AT	161.9 (63.0–187.3)*	20.0 (4.4–46.2)*	−4.115	<.001
	On the 3th d AT	122.7 (64.9–180.9)†	6.3 (0.4–33.0)†	−4.595	<.001
	$\chi^{2}$	9.599	42.247	/	/
	*P* value	.009	<.001	/	/
RDW (%)	BT	15.9 (15.8–16.9)	15.8 (15.2–17.3)	−0.725	.469
	On the 1^st^ d AT	18.5 (17.0–21.3)*	16.2 (15.6–17.1)*	−3.306	0001
	On the 3th d AT	21.9 (20.7–22.1)†	15.7 (15.1–16.4)†	−4.830	<.001
	$\chi^{2}$	11.841	22.586	/	/
	*P* value	.002	<.001	/	/

Compared with BT in the same group.

AT = after treatment, BT = before treatment, DG = death group, PCT = procalcitonin, RDW = distribution width, SG = severe group, SPI = epticaemia premature infants.

* Represents *P* < .05; Compared with the first day AT in the same group.

† Indicates *P* < .05.

### 3.7. Prediction of severe PS using PCT and RDW at different stages

The optimal cutoff values for PCT BT, on the 1st day and the 3rd day AT were 3.474 ng/mL, 29.764 ng/mL, and 3.459 ng/mL, respectively, with sensitivity of 54.7%, 68.9%, and 83.2%, and specificity of 75.8%, 72.1%, and 46.2%, respectively (*P* < .05). The optimal cutoff values for RDW on the 1st and 3rd day AT were 15.649% and 18.299%, respectively, with sensitivity of 95.1% and 29.5%; The specificity was 33.2% and 92.5% respectively (*P* < .05), as shown in Table 7.

**Table 7:** Prediction of severe PS by PCT and RDW at different stages.

Testing index	PCT (ng/mL)			RDW (%)		
Treatment stages	BT	On the 1^st^ d AT	On the 3th d AT	BT	On the 1^st^ d AT	On the 3th d AT
ROC	0.630	0.699	0.674	0.523	0.618	0.628
*P* value	.027	.001	.002	.681	.046	.030
95% CI	0.516–0.745	0.590–0.808	0.567–0.781	0.406–0.641	0.506–0.730	0.514–0.841
Sensitivity (%)	54.7	68.9	83.2	21.3	95.1	33.2
Specificity (%)	75.8	72.1	46.2	92.5	29.5	92.5
Youden index	0.2	0.3	0.2	0.1	0.1	0.2
Optimal boundary value	3.474	29.764	3.459	18.949	15.649	18.299

AT = after treatment, BT = before treatment, PCT = procalcitonin, PS = premature septicaemia, RDW = distribution width, ROC = receiver operating characteristic.

**Figure 3.**
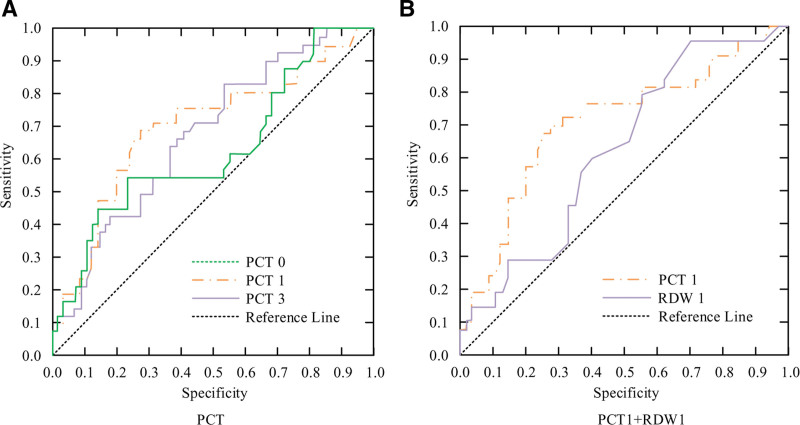
ROC Curve of PCT and RDW predicting PS BT and AT. AT = after treatment, BT = before treatment, PCT = procalcitonin, PS = premature septicemia, RDW = distribution width, ROC = receiver operating characteristic.

From Table 7, the areas under the ROC curve predicted by PCT were 0.630, 0.699, and 0.674, respectively (*P* < .05); The areas under the ROC curve predicted by RDW were 0.618 and 0.628, respectively (*P* < .05). Drawing it into a graph, and the result is shown in Figure 3.

**Figure 4.**
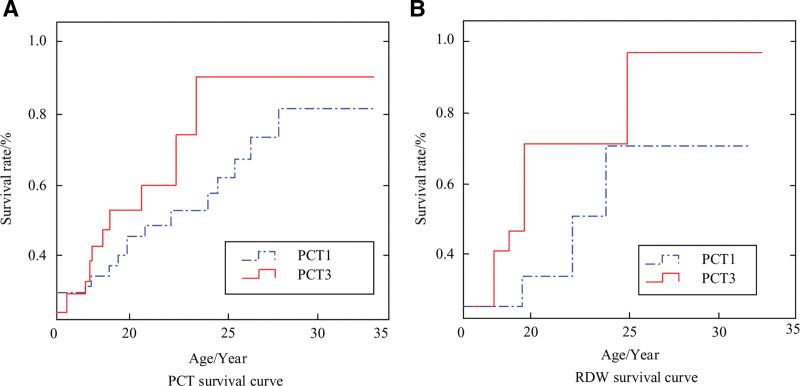
Prediction of recurrence in the prognosis of PS using PCT and RDW. PCT = procalcitonin, PS = premature septicemia, RDW = distribution width.

In the prediction of recurrence in the prognosis of PS using PCT and RDW, the likelihood of recurrence was found to be relatively low (*P* < .05), with statistically significant differences. The results are shown in Figure 4.

### 3.8. Predictive results of PCT and RDW at different stages for death in SPI

The optimal cutoff values for PCT on the 1^st^ and 3rd day AT were 40.594 ng/mL and 64.854 ng/mL, respectively, with sensitivity of 100.0% and 100.0%, and specificity of 73.2% and 87.1% (*P* < .05). The optimal cutoff values for RDW on the 1st and 3rd day AT were 16.649% and 18.449%, respectively, with sensitivity of 100.0% and 100.0%, and specificity of 68.5% and 91.8% (*P* < .05). The results are shown in Table 8.

**Table 8:** Prediction of death in SPI by PCT and RDW at different stages.

Testing index	PCT (ng/mL)			RDW (%)		
Treatment period	BT	On the 1st d AT	On the 3th d AT	BT	On the 1st d AT	On the 3th d AT
ROC	0.626	0.898	0.944	0.569	0.819	0.967
*P* value	.190	<.001	<.001	.467	.001	<.001
95% CI	0.437–0.815	0.820–0.976	0.897–0.992	0.425–0.714	0.723–0.916	0.934–1.000
Sensitivity (%)	50.0	100.0	100.0	90.0	100.0	100.0
Specificity (%)	81.3	73.2	87.1	40.0	68.5	91.8
Youden index	0.2	0.6	0.8	0.2	0.6	0.8
Optimal boundary value	20.449	40.594	64.854	15.649	16.649	18.449

AT = after treatment, BT = before treatment, PCT = procalcitonin, RDW = distribution width, SPI = septicemia premature infants, ROC = receiver operating characteristic.

**Figure 5.**
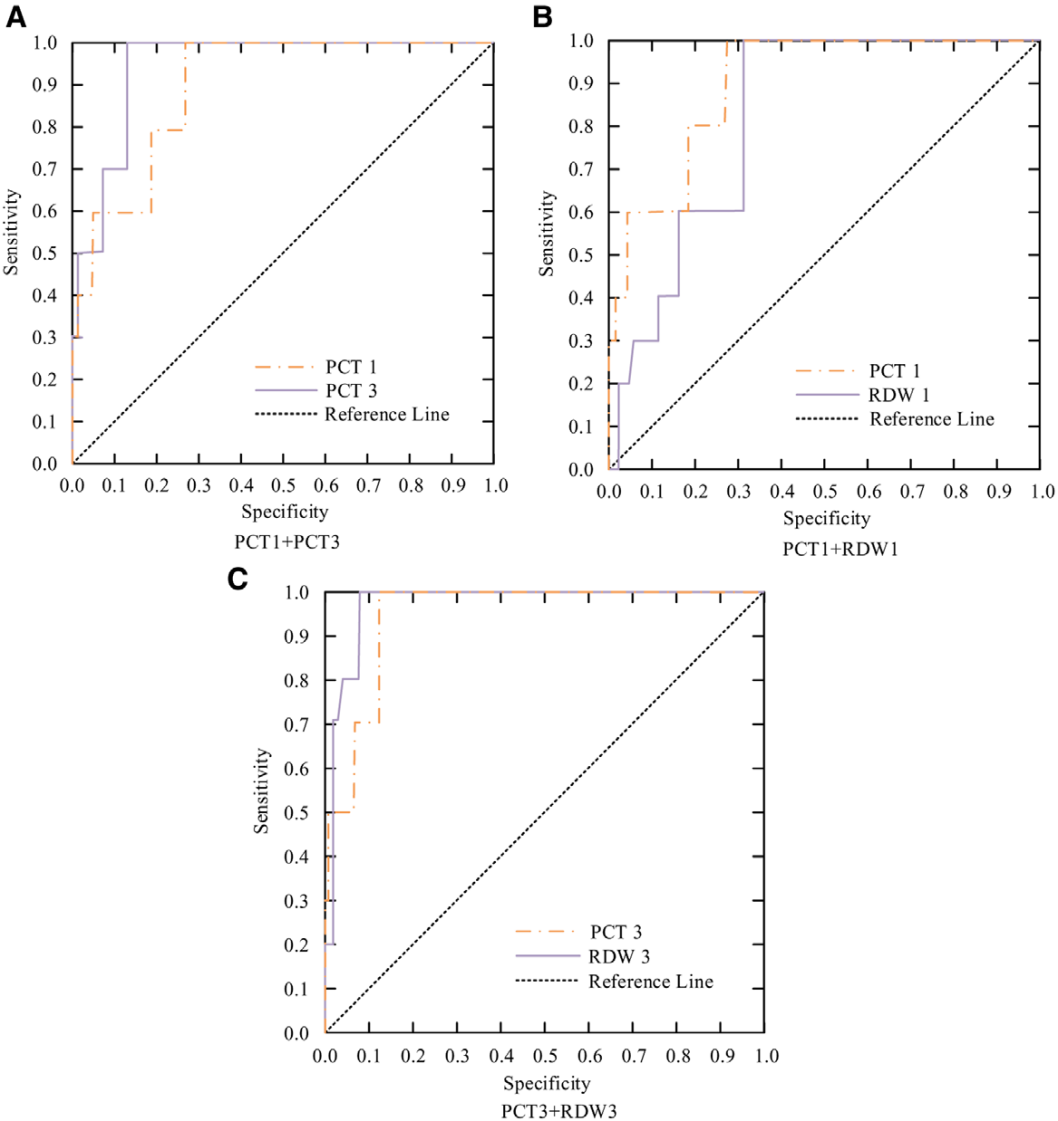
ROC curve of PCT and RDW predicting death in SPI BT and AT. AT = after treatment, BT = before treatment, PCT = procalcitonin, RDW = distribution width, SPI = septicemia premature infants, ROC = receiver operating characteristic.

From Table 8, the areas under the ROC curve predicted by PCT were 0.898 and 0.944, respectively (*P* < .05); The regions under the ROC curve predicted by RDW were 0.819 and 0.967, respectively (*P* < .05). Drawing it into a graph, and the result is shown in Figure 5.

## 4. Discussion

Neonatal septicemia (NS) refers to a systemic infection caused by bacteria or fungi entering the bloodstream during the neonatal period and growing and reproducing in the bloodstream to produce toxins. It is an important cause of neonatal death, complications, and sequelae, with a more significant impact on PI.^[16]^ Most children with NS belong to PI. Due to the immature development of organs in PI and the incomplete formation of cellular immune mechanisms in the body, they are more prone to severe infection symptoms, which poses a significant threat to life.^[17,18]^ When the child has clinical symptoms of PS, auxiliary examinations are needed to determine whether it is diagnosed as PS.^[19]^ Due to the inadequate immune system of PI, they are prone to spread after infection, resulting in a significantly higher incidence of septicemia compared to full-term infants. Moreover, these children’s diseases develop faster and are more likely to develop into septicemia, increasing death and long-term disability rates. NS has a hidden onset and nonspecific clinical symptoms, making it prone to misdiagnosis and missed diagnosis.^[20,21]^ The incidence rate of NS is about 0.1% to 1%, and the death rate is 15% to 50%, which is an important factor causing neonatal death. PCT is a precursor of Calcitonin without hormone activity. The content of PCT in the serum of healthy individuals is low and very stable. PCT is considered as a sensitive indicator of neonatal infectious diseases, with high sensitivity and specificity for bacterial infection, and plays a very important role in the diagnosis of NS. RDW is the most common blood routine test indicator and has been widely used in the diagnosis of various anemia diseases. It is considered an important indicator for predicting the prognosis of severe symptomatic diseases.^[22]^ Research has shown that PCT and RDW have fast and sensitive characteristics in detecting inflammation, and have been widely used in the detection of PS. Therefore, this study will use PCT and RDW for the diagnosis and prognosis evaluation of PS to provide more accurate reference basis for the treatment and prognosis of children.

PCT is an immune regulatory protein, and the content of PCT in the serum of healthy individuals is very low. After bacterial infection, stimulated by inflammatory factors and other factors, its concentration will rapidly increase within 2 to 6 hours and reach its peak within 12 to 48 hours. The time of its rise and peak appearance can well indicate the occurrence of bacterial infection. The PCT in serum is related to the adverse prognosis of septicemia, and its dynamic changes can be used as an index to determine the treatment effect and prognosis of septicemia. PCT will have a physiological increase 3 days after birth, generally below 0.5 ng/mL in 48 to 72 hours, and will drop to the level of adults in 5 to 7 days, which is 0.05 ng/mL. So, after 3 days of birth, the original changes in PCT of newborns will not have any impact. The study by Hegamyer E et al^[23]^ showed that compared to other experimental indicators, PCT had greater advantages in evaluating infection and other aspects. Klymenko T and other experts found that in both the SG and DG, the concentration of PCT in the serum was significantly increased, which may be due to the inflammatory cascade reaction caused by neonatal bacterial infections.^[24]^ The higher the PCT content, the more severe the situation of NS and the higher the death rate. The results of this study also confirmed this point. The PCT of the SG BT, on the first and third days AT, were 3.8 ng/mL, 43.2 ng/mL, and 26.2 ng/mL, respectively; The PCT in the MG BT, on the first and third days AT, were 1.5 ng/mL, 17.2 ng/mL, and 24.7 ng/mL, respectively. The PCT in the DG were 161.9 ng/mL, 122.7 ng/mL, and 12.2 ng/mL on the first and third days AT and BT, respectively; The PCT in the SG were 20.0 ng/mL, 6.3 ng/mL, and 1.7 ng/mL on the first and third days AT and BT. The optimal cutoff values for PCT on the first and third days AT were 40.594 ng/mL and 64.854 ng/mL, respectively, with sensitivity of 100.0% and 100.0%, and specificity of 73.2% and 87.1% (*P* < .05). This result is consistent with the research content.

RDW is an index that reflects the dispersion of red blood cell size, which belongs to the blood routine and is closely related to the prognosis of many diseases. Experts such as Xanthopoulos A have found that RDW is a powerful predictive indicator in patients.^[25]^ Dai L et al^[26]^ found that in critically ill patients with internal medicine, the higher the RDW, the higher the death rate. Therefore, RDW can be used as an indicator to determine the severity of a patient’s condition. The results of this study also confirmed that the RDW in the SG were higher than those in the MG. The RDW in thed eath group were 21.9%, 18.5%, and 15.9% on the 3rd and 1st day AT and BT, respectively; The RDW in the SG on the first day, BT, and on the third day AT were 16.2%, 15.8%, and 15.7%, respectively. The optimal cutoff values for RDW on the first and third days AT were 16.649% and 18.449%, respectively, with sensitivity of 100.0% and 100.0%, and specificity of 68.5% and 91.8%, respectively (*P* < .05). This result is consistent with the research content.

From the above research, PCT and RDW can respond sensitively to the severity of PS, and their changes in content can better reflect the efficacy and prognosis of PS. In SPI, the higher the PCT and RDW, the poorer the prognosis; PCT has higher predictive value than RDW in predicting the prognosis of PS. Given the high death rate and rapid progression of PS, dynamic monitoring of serum PCT and RDW concentrations was conducted. This can assist clinical physicians in early diagnosis and treatment of PS, which will improve the symptoms of the child and be beneficial for prognosis; More importantly, this can also reduce the death rate, thereby reducing the occurrence of septicemia. The number of samples in this study is relatively small, and further research will be conducted by increasing the number of samples in the future.

## Author contributions

**Conceptualization:** Huafen Xu, Dong Cai, Lixia Zhou.

**Data curation:** Huafen Xu, Dong Cai, Lixia Zhou.

**Formal analysis:** Huafen Xu, Dong Cai.

**Investigation:** Huafen Xu, Dong Cai, Lixia Zhou.

**Methodology:** Huafen Xu, Dong Cai, Lixia Zhou.

**Supervision:** Lixia Zhou.

**Writing – original draft:** Huafen Xu.

**Writing – review & editing:** Huafen Xu.
